# Metabarcoding of ichthyoplankton communities associated with a highly dynamic shelf region of the southwest Indian Ocean

**DOI:** 10.1371/journal.pone.0284961

**Published:** 2023-04-27

**Authors:** Ashrenee Govender, Sean T. Fennessy, Sean N. Porter, Johan C. Groeneveld

**Affiliations:** 1 Oceanographic Research Institute, Durban, South Africa; 2 School of Life Sciences, University of KwaZulu-Natal, Durban, South Africa; MARE – Marine and Environmental Sciences Centre, PORTUGAL

## Abstract

Drifting fish eggs and larvae (ichthyoplankton) can be identified to species using DNA metabarcoding, thus allowing for post hoc community analyses at a high taxonomic resolution. We undertook a regional-scale study of ichthyoplankton distribution along the east coast of South Africa, focused on the contrasting environments of the tropical Delagoa and subtropical Natal Ecoregions, and on exposed and sheltered shelf areas. Zooplankton samples were collected with tow nets at discrete stations along cross-shelf transects (20–200 m depth) spaced along a latitudinal gradient that incorporates a known biogeographical boundary. Metabarcoding detected 67 fish species, of which 64 matched prior distribution records of fishes from South Africa, with the remaining three known from the Western Indian Ocean. Coastal, neritic and oceanic species were present, from epi- and mesopelagic to benthopelagic and benthic adult habitats. By family, Myctophidae (10 species), Carangidae, Clupeidae, Labridae (each with 4 species) and Haemulidae (3 species) were most speciose. Ichthyoplankton community composition varied significantly with latitude, distance to coast, and distance to the shelf edge. Small pelagic fishes had the highest frequency of occurrence: *Engraulis capensis*, *Emmelichthys nitidus* and *Benthosema pterotum* increased in frequency towards the north, whereas *Etrumeus whiteheadi* increased towards the south. Chub mackerel *Scomber japonicus* accounted for most variability related to distance from the coast, whilst African scad *Trachurus delagoa* correlated with distance to the shelf edge. Dissimilarity between communities in the Delagoa and Natal Ecoregions was 98–100%, whereas neighbouring transects located within the sheltered KwaZulu-Natal Bight had lower dissimilarity (56–86%). Onshore transport of ichthyoplankton by Agulhas Current intrusions plausibly explained the abundance of mesopelagic species over the shelf. Metabarcoding followed by community analysis revealed a latitudinal gradient in the ichthyoplankton, associations with coastal and shelf-edge processes, and evidence of a spawning area in the sheltered KwaZulu-Natal Bight.

## Introduction

DNA metabarcoding can simultaneously identify all the species present in taxonomically complex samples, by combining high throughput sequencing (HTS) technology with barcode reference data which link individual species to specific sequences [1, 2]. Detected species can then be used in biodiversity indices, monitoring of essential biodiversity variables and in community ecology [3, 4]. Metabarcoding is particularly well suited to uncovering ichthyoplankton (fish eggs and larvae) diversity, because such communities are comprised of numerous organisms at different life stages (eggs, larvae, juveniles, and adults) which cannot always be identified visually [5, 6]. Fishes are well-represented in DNA barcode reference libraries [7–9] and therefore most species present in ichthyoplankton samples can be identified through metabarcoding analysis [10–12].

Ichthyoplankton assemblages in marine pelagic environments are primarily influenced by species-specific fish spawning locations (e.g., coastal, neritic, or oceanic) and reproductive seasons [13]. Assemblages are further influenced by water movements (currents, eddies, wind drift), physical-chemical parameters (temperature, salinity, dissolved oxygen, pH) [14–19] and vertical migration behaviour [20, 21]. Ichthyoplankton diversity and distribution patterns can provide information on fish spawning areas, reproductive seasons, migration routes, ecological connectivity, and recruitment processes, all of which are important in guiding management and conservation effort [10, 22].

Past research on ichthyoplankton in eastern South Africa (KwaZulu-Natal province; KZN) focussed on estuarine nursery areas, harbours and the surf zone, relying on visual identification methods to determine ichthyoplankton composition, relative abundance and seasonality [23–29]. Hutchings et al. (2002) showed that the shallow offshore KZN Bight provides a fish spawning habitat and nursery ground [13]. It was proposed that larval survival in the bight would be enhanced through enrichment, retention and concentration mechanisms [30–32]. Oceanographic surveys undertaken in the Agulhas Current region during the early 1990s provided information on ichthyoplankton occurring further offshore, over the deeper KZN shelf and upper slope [14, 33–36].

The advent of metabarcoding now allows for more accurate and rapid determination of ichthyoplankton species present in plankton samples, facilitating finer-scale community analyses to address ecological questions [18, 19, 37]. We used metabarcoding to identify ichthyoplankton species collected across two distinct Ecoregions, encompassing the bight and the exposed shelf on the east coast of South Africa, to investigate variation in community composition according to spatially structured environmental attributes. Specific aims were to: (1) generate a presence-absence matrix of ichthyoplankton species based on metabarcoding analyses and a post hoc verification process, (2) test for the presence of ichthyoplankton species from a wide range of habitats and geographic distribution ranges, and (3) explore patterns in ichthyoplankton community composition in relation to several prominent environmental features such as tropical and subtropical Ecoregions, a sheltered nursery area in the KZN Bight, and exposure to the strong western-boundary Agulhas Current and inshore coastal processes such as riverine input.

## Materials and methods

### Study area and field sampling

**Fig 1:**
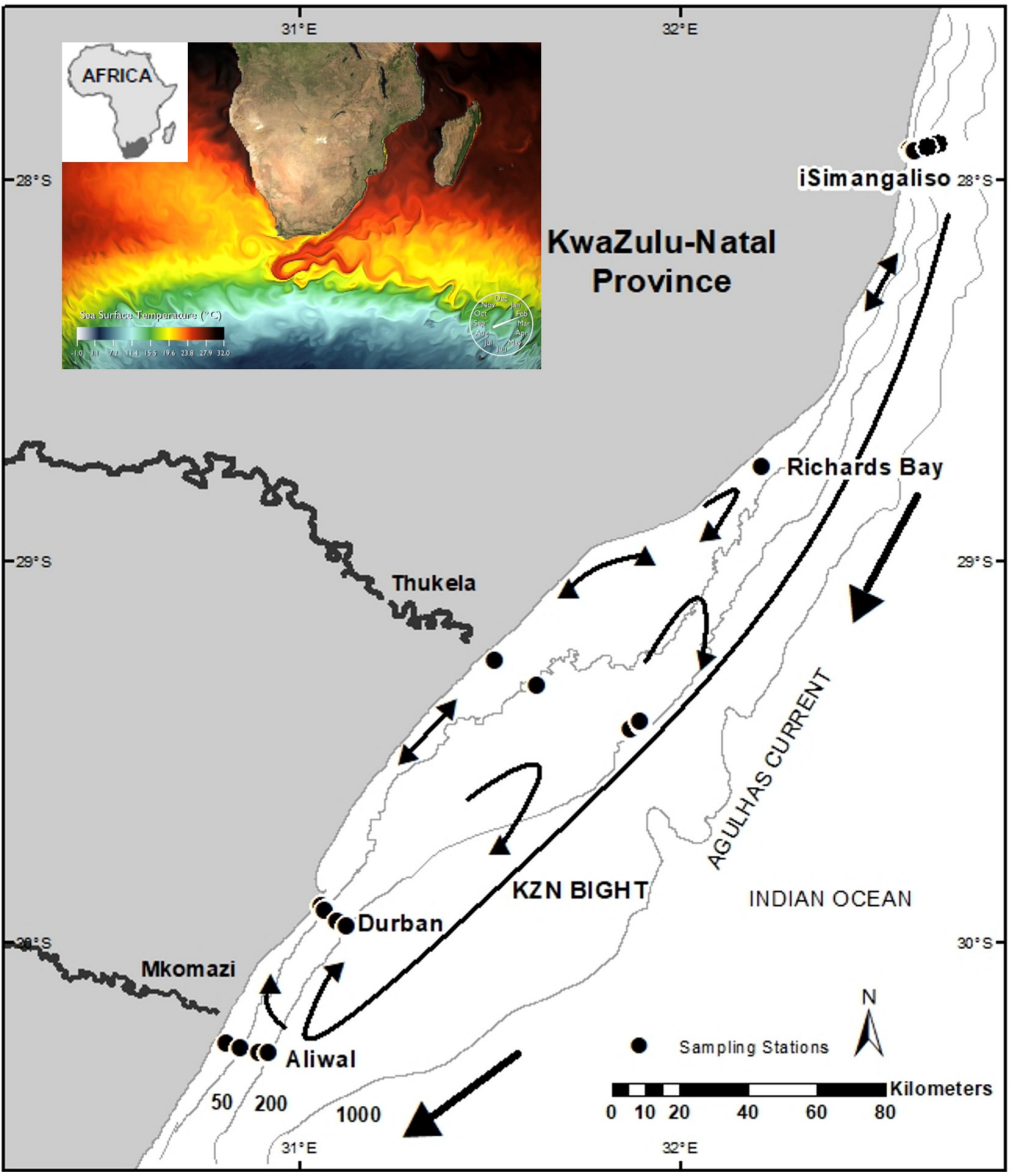
Location of cross-shelf transects at iSimangaliso, Richards Bay, Thukela, Durban and Aliwal, with sampling stations at 20, 50 100 and 200 m bottom-depth soundings. The proximity of the Agulhas Current to the coast and eddies / counter-currents over the sheltered KwaZulu-Natal Bight were derived from [42]. The satellite image of sea surface temperature (inset) shows the transport of tropical waters southwards along the coast by the Agulhas Current (image obtained from NOAA).

The east coast of South Africa forms the southwestern extreme of the Western Indian Ocean (WIO) (Fig 1) and is strongly influenced by the western-boundary Agulhas Current [38]. The current flows south-westwards along the seaward edge of a narrow continental shelf which slopes down steeply after reaching 100 m depth, some 3–11 km from the coast, except in the broader KZN Bight, a coastal offset of 160 km long with a maximum shelf-width of 45 km situated between Durban and Richards Bay [39]. The Delagoa Ecoregion, commencing 90 km north of Richards Bay, is characterised by relatively warmer tropical waters low in turbidity and chlorophyll, whilst the Natal Ecoregion to the south is subtropical and experiences significantly higher riverine input which contributes to contrastingly higher levels of nearshore turbidity and chlorophyll, especially in the KZN Bight [40, 41].

Ichthyoplankton was sampled with plankton nets towed behind a research vessel at sampling stations along cross-shelf transects at iSimangaliso and Richards Bay (August 2018), Thukela (September 2018), Durban (August 2018 and 2019) and Aliwal (November 2018) (Fig 1). Discrete sampling stations along the transects were located at 20, 50, 100 and 200-m depth isobaths. Three net types were used at each station to maximise the number of species caught: a plankton ring-net (500 μm mesh; 0.8 m ring diameter; towed horizontally at <5 m depth); manta net (500 μm mesh; towed at the surface); and a WP2 net (200 μm; 0.55 m ring diameter; lowered to 10 m above the seafloor and hauled vertically). Surface nets were towed for 5 minutes, at a ground speed of 2–3 knots. Each tow constituted a sample (S1 Table; n = 88). Samples were washed from the cod-end into a jar with 95% ethanol and stored at -20°C at the Oceanographic Research Institute. Inclement weather prevented sampling at the 200 m station at iSimangaliso and at the 50, 100 and 200 m stations at Richards Bay.

### Laboratory processing of samples

#### Extraction of genomic DNA

Individual tow net samples (S1 Table) were homogenized for 45 s using a consumer blender (Defy PB7354X, 350 W and 22,000 rpm) [43]. Three subsamples per homogenate were taken to increase sequencing depth and improve diversity estimates. Each subsample (10 ml of zooplankton) was centrifuged at 1200 x g for 1 min, repeated to remove excess ethanol; thereafter 40 mg of tissue was transferred to a sterile tube. The remaining DNA extraction process was carried out using the Qiagen DNeasy Blood and Tissue Kit following the manufacturer’s standard protocol which was slightly modified by adding 180 μl ATL buffer and 40 μl proteinase K to the tissue for overnight lysis at 56°C. The extracted DNA from the three subsamples per homogenate were then pooled (n = 88) and stored at −20°C. Laboratory blanks were used consistently during DNA extractions to test for contamination.

#### PCR amplification, library preparation and high-throughput DNA sequencing

Polymerase chain reactions (PCRs) were performed in triplicate to minimize bias and amplification errors. Universal primers (313–319 bp) [44, 45] and a primer cocktail for fish (313–319 bp) [46] were used for PCR to amplify fragments of the mitochondrial COI gene region (see [47] for primer combinations). PCRs (25 μl) contained 0.25 μl Q5 High-Fidelity DNA Polymerase (0.02 U μl−1, New England BioLabs), 5 μl Q5 reaction buffer (1×), 5 μl Q5 high GC enhancer (1×), 0.5 μl dNTPs (10 mm of each), 1 μl forward and reverse primers (5 μm), 1 μl template DNA (10 ng μl−1), 2 μl additional MgCl2 (25 μm), 2 μl bovine serum albumin (BSA; 1 mg ml) and nuclease-free water. Thermal cycling consisted of initial denaturation at 98°C for 30 s, and 25 cycles at 98°C for 10 s each, annealing at 46°C for 30 s, extension at 72°C for 30 s, and a final extension at 72°C for 4 min. All PCRs included a no-template negative control. PCR products were visualized on a 1% (w/v) TBE agarose gel containing 0.02% ethidium bromide (EtBr). Amplicon size was determined using a 100-bp molecular weight marker (Solis Biodyne). The triplicate PCR products for each of the primer sets (universal and fish) were pooled and quantified using a Qubit 2.0 Fluorometer (Life Technologies, California, USA). The pooled products were consolidated into a single sample for each tow-net haul, to create 88 libraries with equimolar concentrations (5 ng/μl). Illumina Miseq sequencing was performed at the KZN Research and Innovation Platform (KRISP, South Africa) following the protocols outlined in [43].

#### Taxonomic assignment of Amplicon Sequence Variants (ASVs) and cross-referencing with occurrence records

The dada2 algorithm [48] implemented in QIIME2 v. 2019.10 [49] was used for quality control checks, chimera removal, filtering, trimming of primers, truncation of forward and reverse reads and merging of paired-end reads into amplicon sequence variants (ASVs). The ASVs were queried against BOLD systems version 4 (https://www.boldsystems.org) and NCBI GenBank (https://www.ncbi.nlm.nih.gov/genbank/; query cover > 80%) in January to March 2022. Thereafter, ASVs for ichthyoplankton only were filtered out for analyses. ASVs assigned to the same ichthyoplankton species were merged manually using MS Excel. A 97% sequence identity threshold was used for taxonomic assignment to species level.

Fish species detected by metabarcoding were further validated by cross-referencing with occurrence records obtained from online databases such as the World Register of Marine Species (WoRMS; https://www.marinespecies.org), the Ocean Biodiversity Information System (OBIS; https://obis.org) and the Global Biodiversity Information Facility (GBIF; https://www.gbif.org). In cases where more than one species name was associated with a GenBank or BOLD record, these species names were cross-checked, including use of knowledge of WIO ichthyofauna (SF), as well as consultation with other species experts. In almost all cases (*Chromis* and *Umbrina* excepted), it was apparent that a single species was present, and that misnomers were a result of reference sequences of closely related species originating from other ocean regions.

#### Exploratory analysis

The presence of ichthyoplankton originating from a broad range of known adult habitats, ocean zones and geographic distribution ranges was explored. Adult habitats of the identified ichthyoplankton species were categorized as benthic-reef, benthic-soft sediment, benthopelagic, epipelagic and mesopelagic based on the literature [50, 51] or consultation with experts. Ocean zones were categorized as coastal (< 50 m deep), neritic (shelf > 50 m deep and upper continental slope) and oceanic (offshore of the slope). Species distribution ranges were categorized hierarchically as endemic to the Southwest Indian Ocean (SWIO; eastern South Africa, Mozambique Channel and tropical WIO south of the equator), Western Indian Ocean (WIO; the former, but including the Red Sea, Arabian Sea, and Northwest Indian Ocean), Indo-West Pacific (IWP) and circumglobal. Spatial patterns in species diversity, in the form of the total number of species detected per sample, were graphically indicated with interpolation using inverse-distance weighting at a 1-km spatial resolution and a longitude-orientated projected coordinate system employing the WGS84 reference ellipsoid.

#### Ichthyoplankton community analysis

A species-level matrix of ichthyoplankton presence-absence data by sample was constructed as input to a multivariate analysis with explanatory variables for station, transect (iSimangaliso, Richards Bay, Thukela, Durban, Aliwal), latitude, longitude, depth isobath of station (20, 50, 100 or 200 m contours), distance from the coast and from the shelf-edge (defined as the 200-m depth contour), net type (ring-, manta- and WP2 nets) and date of sampling (converted to serial date for analysis).

The Sorenson’s similarity index for presence-absence data was computed among samples [52]. Variation in plankton community composition according to the explanatory variables was investigated using a sequential multivariate permutational analysis of covariance (PERMANCOVA) [53]. Relationships between pairs of continuous explanatory variables were investigated prior to this for high (>0.9) correlations that would indicate redundancy, using Pearson correlation. Longitude was removed as it was highly correlated (r = 0.97) with latitude. Serial date and net type were considered nuisance variables [54] as sampling could not be done simultaneously and not all three net types could be used at each station. As such, these two nuisance variables were fitted first in the model, so that their effects could be removed prior to fitting latitude, distance from coast, distance from shelf-edge, depth isobath, transect and station nested in transect. Transect was considered a fixed effect and station a random effect. The covariates of interest were fitted in descending order of their spatial range: latitude, distance from coast, distance from shelf-edge and isobath. The analysis was run with type I sequential sums of squares with 9999 Monte-Carlo permutations of residuals under a reduced model.

Constrained multi-dimensional scaling ordinations of the first and second pairs of axes derived from a canonical analysis of principle coordinates analysis were used for graphical display of the samples and their relationships with the environmental and ichthyoplankton variables [55, 56]. Vectors of the correlations of the significant environmental variables of interest along with those species with relatively high correlations with the respective canonical axes of each plot were overlaid onto the two ordinations. Lastly, a similarity percentage breakdown analysis (SIMPER) according to transect was done to indicate which species were contributing to similarities and differences at this level [57]. All statistical analyses were done with the software program PERMANOVA+ for PRIMER 7.0.21 [58].

## Results

### High-throughput sequencing results

Sequencing was efficient with minimal filtering needed when merging the paired-end reads for all 88 zooplankton libraries. A total of 9.4 million read counts were consolidated into 346,748 merged reads (S2 Table). A total of 1726 sequences were available for analysis across all groups amplified, with 219 sequences available for ichthyoplankton. The 219 sequences were collapsed into 90 ASVs of which 67 (74%) could be matched to a species level with >97% sequence similarity to sequences on BOLD or GenBank.

### Species diversity identified by metabarcoding

**Fig 2:**
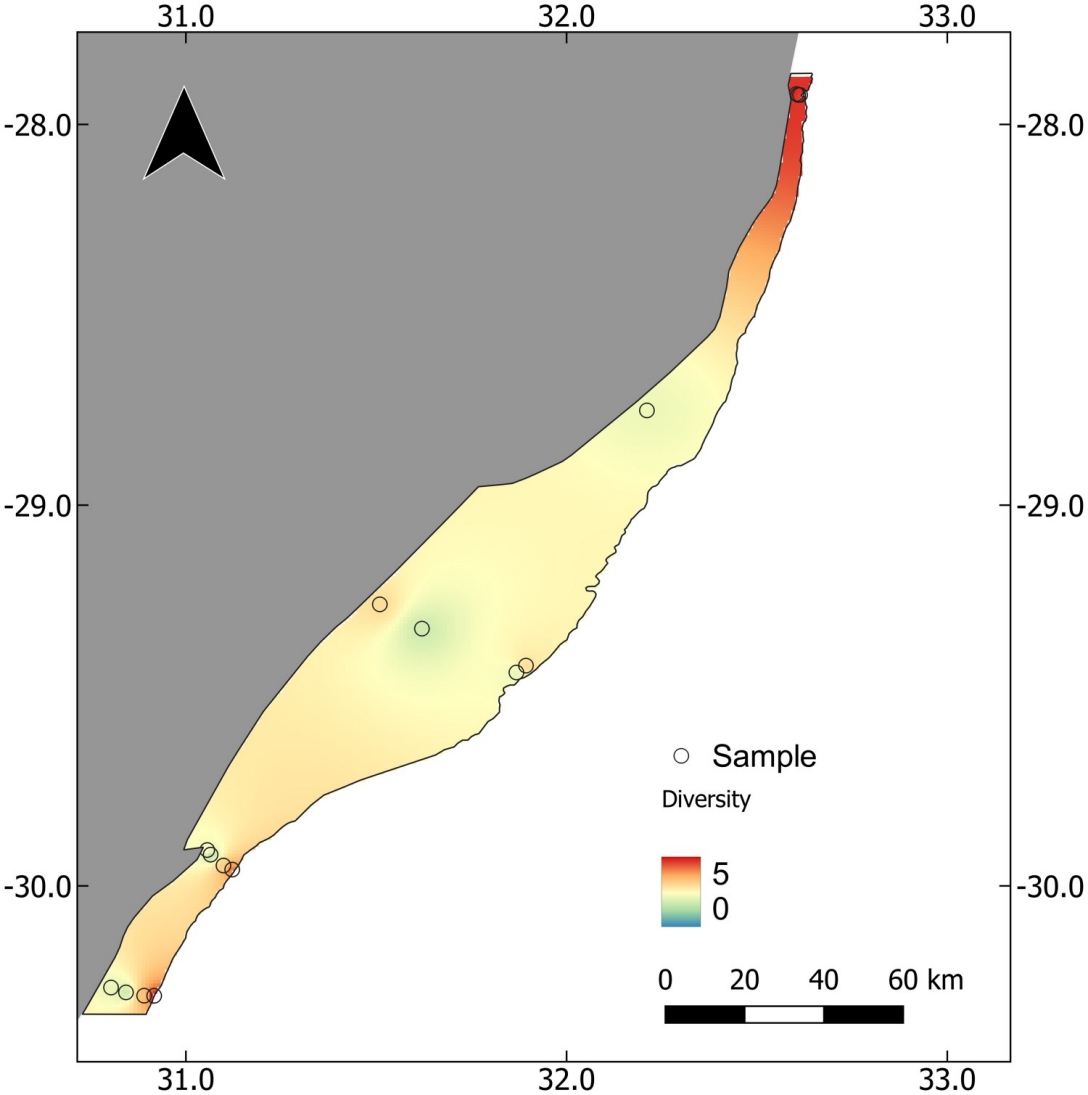
Ichthyoplankton diversity on the east coast of South Africa derived from inverse-distance weighted interpolation of the number of species detected in each sample.

Metabarcoding detected a total of 21 ichthyoplankton orders which comprised 36 families, 58 genera and 67 species at a 97% sequence similarity threshold (S3 Table). The orders Myctophiformes and Perciformes (10 spp. each) were best represented, followed by Clupeiformes (6 spp.) and Scombriformes (5 spp.). By family, Myctophidae (10 spp.), Carangidae (4 spp.), Clupeidae (4 spp.), Labridae (4 spp.) and Haemulidae (3 spp.) were most speciose. Of the 67 species detected, 64 (95.5%) matched distribution records from South Africa, with the remaining 3 species (4.5%) not previously recorded from South Africa but known from the WIO region (S3 Table). Average ± SD species diversity per sample over the study region was 3.0 ± 2.0 and range from 0 to 9. Diversity was higher in the north (closer to the tropics) and along the shelf edge (Fig 2).

**Fig 3:**
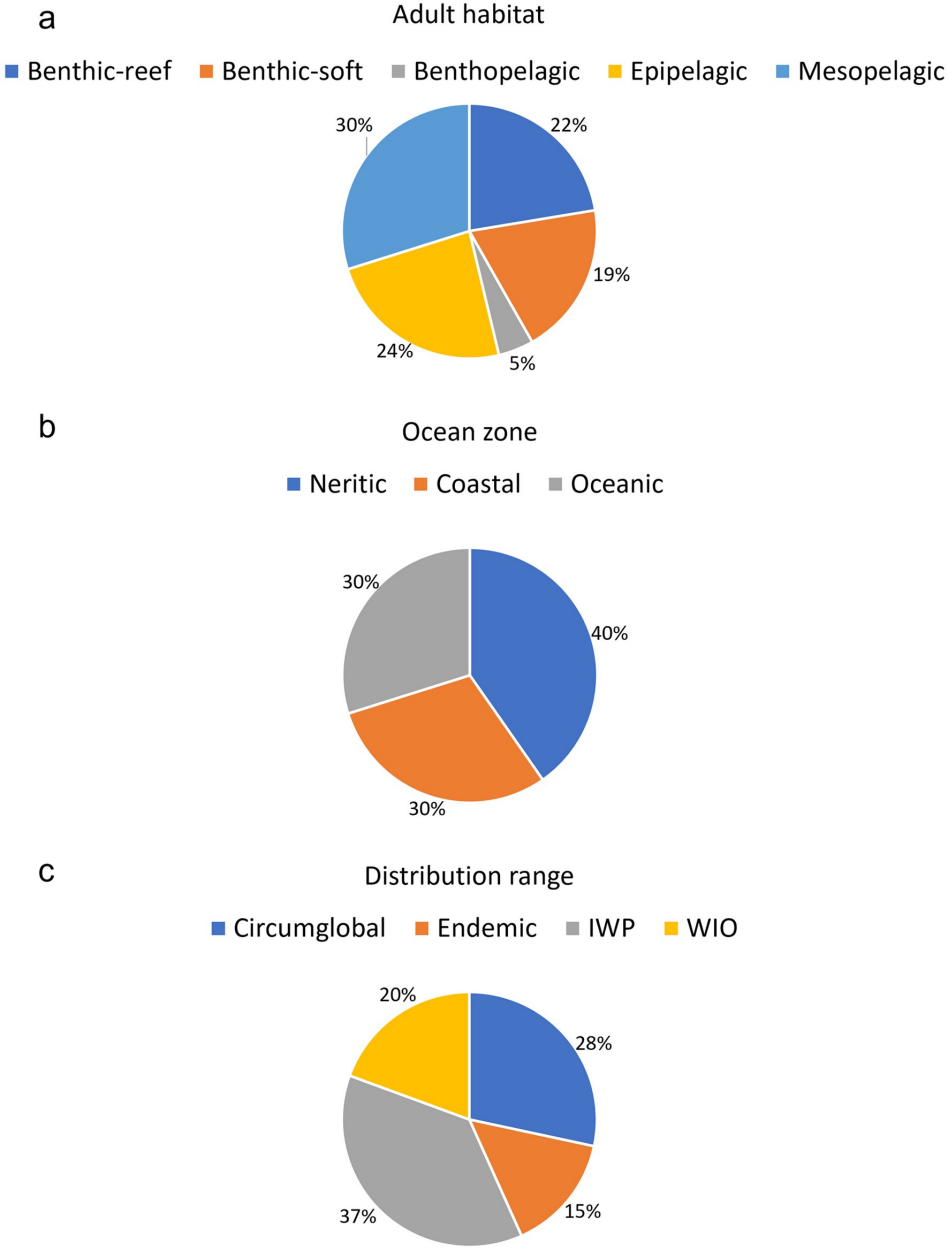
Proportional occurrence of ichthyoplankton species detected by metabarcoding of zooplankton, categorized according to existing data of (a) adult habitats, (b) ocean zone and (c) geographical distribution ranges (WoRMS; GBIF; OBIS).

Ichthyoplankton species originated from a variety of adult habitats including mesopelagic (30% of all detected species), epipelagic (24%), benthic (41% for reef and soft sediment habitats combined) and benthopelagic habitats (5%) (S3 Table; Fig 3a). Species classified as coastal (30%), neritic (40%) and oceanic (30%) were all well-represented in samples (Fig 3b). Species endemic to the SWIO (15%), species from the broader WIO region (21%), those with an IWP origin (36%) and with a circumglobal distribution (28%) were all present in the metabarcoding detections (Fig 3c). No false positive species (present in metabarcoding outputs but unlikely to occur in the sampled region) were observed, confirming that the 97% sequence similarity threshold used was robust for fish species identification using COI.

### Community analysis

**Fig 4:**
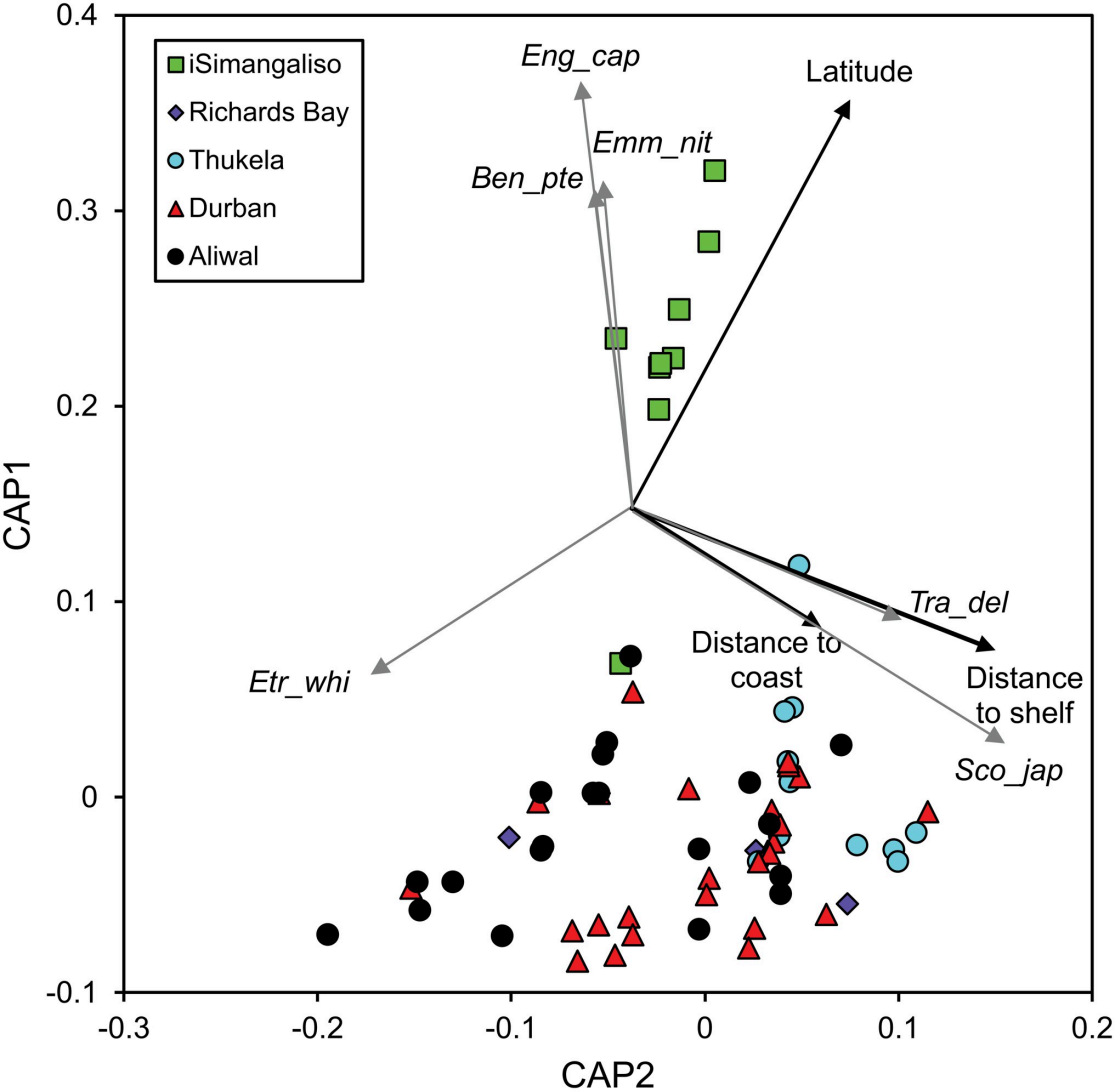
Constrained multidimensional scaling ordination derived from a canonical analysis of principle coordinates of ichthyoplankton samples, plotted according to transect. Vectors of the correlations of the significant environmental variables of interest (black) along with vectors (grey) of those species (abbreviated, see below) with correlations >0.3 with the first two canonical axes are overlaid onto the ordination. The ordination accounted for 44% of the variation and the first and second squared canonical correlations were 0.65 and 0.36, respectively. Ben_pte = *Benthosema pterotum*, Emm_nit = *Emmelichthys nitidus*, Eng_cap = *Engraulis capensis*, Etr_whi = *Etrumeus whiteheadi*, Sco_jap = *Scomber japonicus*, Tra_del = *Trachurus delagoa*.

A summary of the environmental covariates associated with the community analysis are provided in S4 Table. The multivariate analysis of covariance found that ichthyoplankton community composition varied significantly with latitude (*P*_(Monte-Carlo)_ = 0.0001), distance to coast (*P*_(Monte-Carlo)_ = 0.023), distance to the shelf edge (*P*_(Monte-Carlo)_ = 0.0122) and transect (*P*_(Monte-Carlo)_ = 0.0141), although post hoc analyses of transect did not find significant differences (*P*_Monte-Carlo_ ≥ 0.5539). Depth isobath (*P*_Monte-Carlo_ = 0.0719) and station nested within transect were also not significant (*P*_Monte-Carlo_ = 0.1817), as were none of the interaction terms of interest (*P*_Monte-Carlo_ > 0.05). The constrained multi-dimensional scaling ordination accounted for 44% of the variation in the first two canonical axes (Fig 4). The first and second squared canonical correlations were relatively modest at 0.65 and 0.36, respectively. The analysis indicated that ichthyoplankton composition changed along a latitudinal gradient on axis 1 with the highest canonical eigenvector of -0.836, and according to distance to the shelf edge and distance to the coast on axis 2 with canonical eigenvectors of 0.750 and 0.393, respectively. The frequency of *Engraulis capensis*, *Emmelichthys nitidus* and *Benthosema pterotum* increased northwards with correlations with axis 1 of -0.876, -0.673, -0.649, respectively, whereas *Etrumeus whiteheadi* showed the opposite trend with a correlation of 0.343. The frequency of *Trachurus delagoa* increased with increasing distance to the shelf-edge with a correlation of 0.465 with axis 2, while *Scomber japonicus* increased in frequency with increasing distance to the coast with a correlation of 0.643 with axis 2.

**Fig 5:**
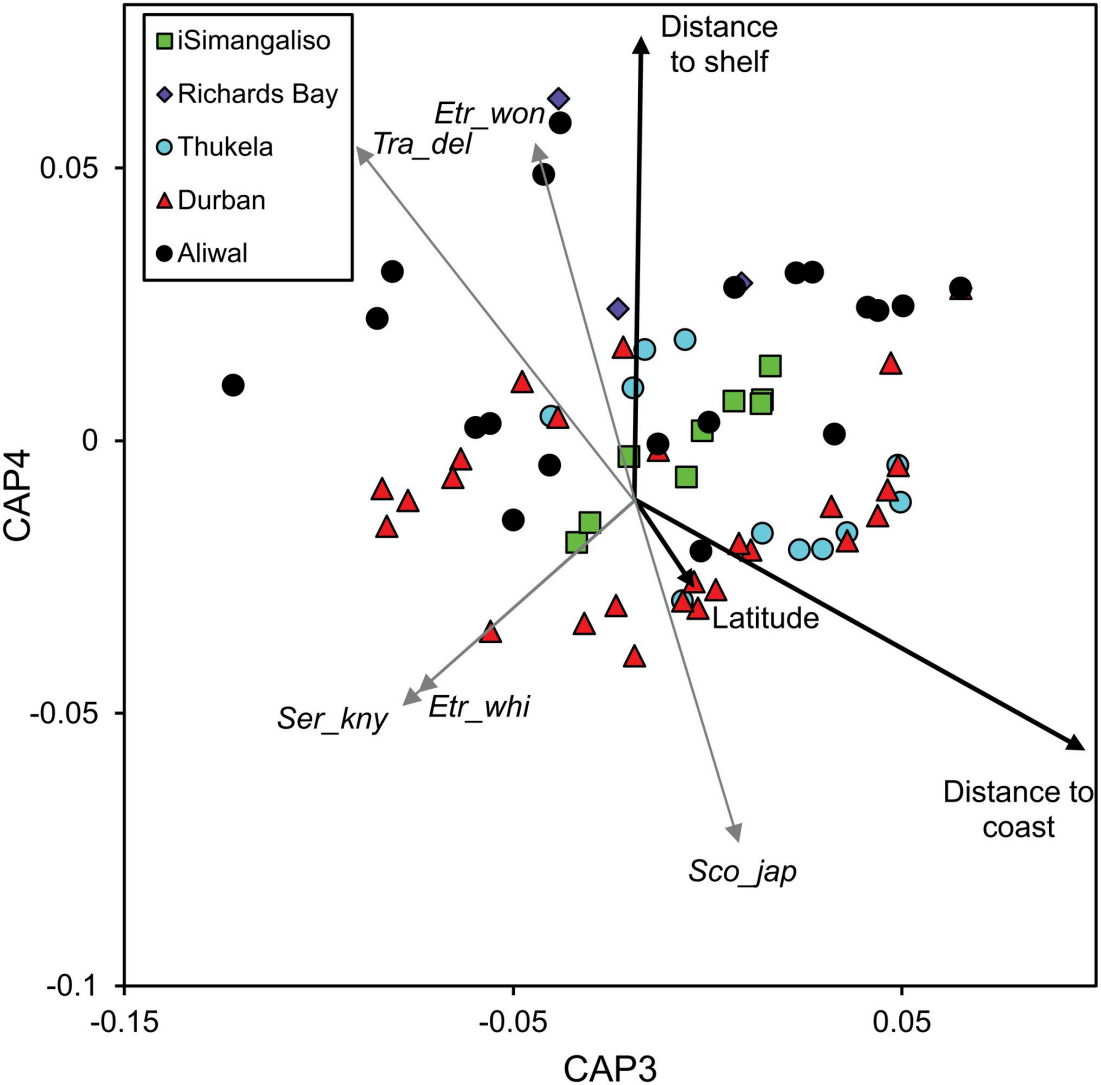
Constrained multidimensional scaling ordination derived from a canonical analysis of principle coordinates of ichthyoplankton samples, plotted according to transect. Vectors of the correlations of the significant environmental variables of interest (black) along with vectors (grey) of those species (abbreviated, see below) with correlations >0.4 with the third and fourth canonical axes are overlaid onto the ordination. The ordination accounted for 26% of the variation and the third and fourth squared canonical correlations were 0.17 and 0.04, respectively. Ben_pte = *Benthosema pterotum*, Emm_nit = *Emmelichthys nitidus*, Eng_cap = *Engraulis capensis*, Etr_whi = *Etrumeus whiteheadi*, Sco_jap = *Scomber japonicus*, Tra_del = *Trachurus delagoa*.

The third and fourth canonical axes explained a further 26% of the variation in community composition, largely attributable to distance to the coast and distance to the shelf edge, with canonical eigenvectors of 0.744 and 0.572, respectively (Fig 5). The frequency of *T*. *delagoa*, *Serranus knysnaensis* and *E*. *whiteheadi* decreased with increasing distance to the coast, with correlations of -0.699, -0.604 and -0.572 with axis 3, respectively. The frequency of *S*. *japonicus* decreased with increasing distance to the shelf edge with a correlation of -0.564 with axis 4, whereas *Etrumeus wongratanai* and *T*. *delagoa* increased with increasing distance to the shelf edge, with correlations of 0.421 and 0.416 with axis 4, respectively.

**Fig 6:**
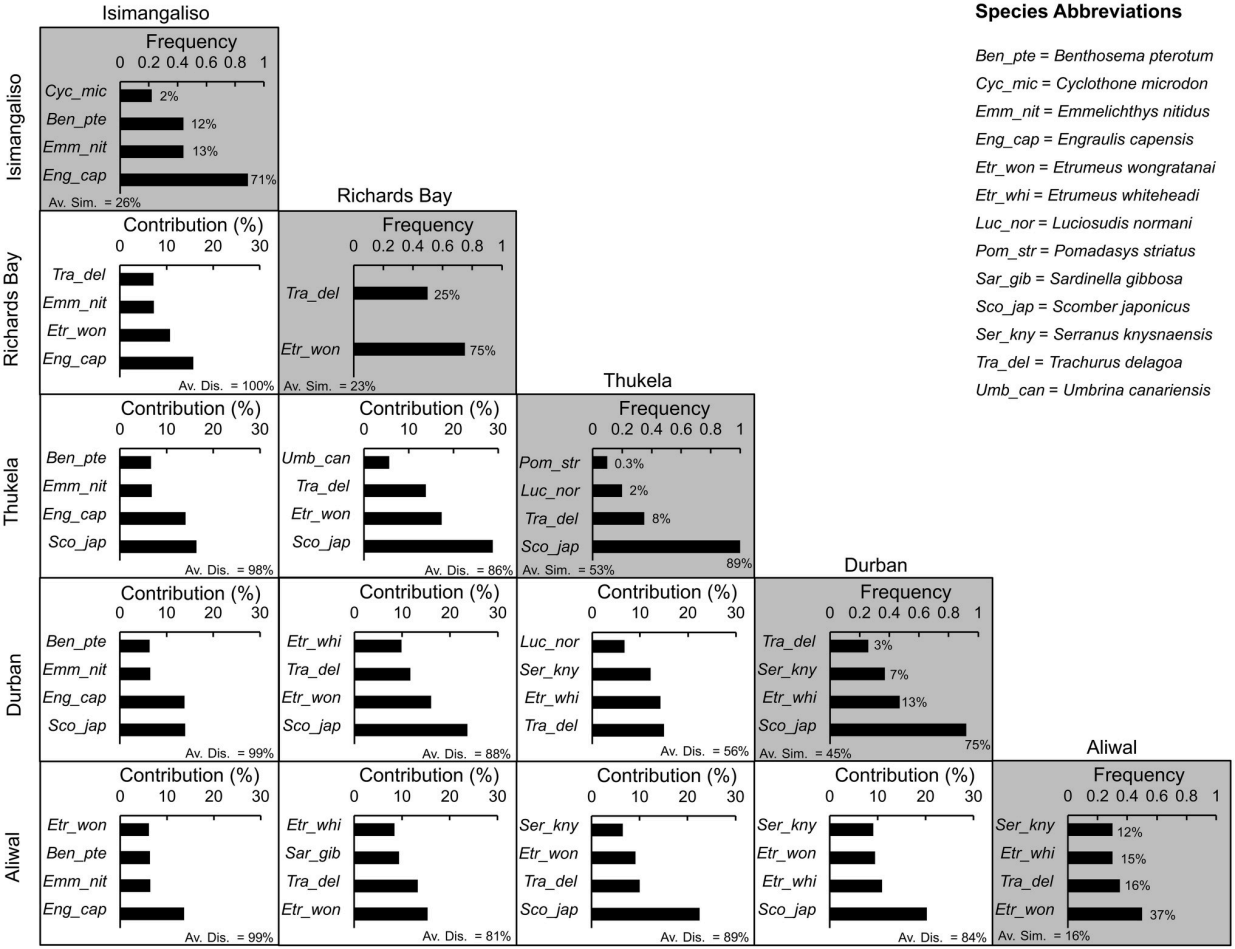
Similarity percentage breakdown (SIMPER) analysis of ichthyoplankton presence-absence data according to transect (north to south). The grey blocks indicate the top-four (most characteristic) species in each transect based on their frequency of presence relative to the total number of samples in that transect, along with the species’ contributions (%) towards the average similarity (Av. Sim.) of that transect. The white blocks represent the top-five species which had the largest contribution (%) to the average dissimilarity (Av. Dis.) between transects.

Similarity percentage breakdown analysis found that differences between the ichthyoplankton communities of iSimangaliso and all the other transects were the most marked, with dissimilarities ranging from 98–100% (Fig 6). The Durban and Thukela transects had the lowest dissimilarity of 56%. Within a transect, Aliwal had the lowest similarity of 16% and Thukela the highest at 53%. *Scomber japonicus* was the most characteristic species at the Durban (75%) and Thukela (89%) transects, whereas *E*. *wongratanai* was the most characteristic species at the Aliwal (37%) and Richards Bay (75%) transects. At the iSimangaliso transect, *E*. *capensis* contributed the most (71%) to the average similarity and played a key role in distinguishing this transect from all other transects, contributing 14–16% to dissimilarity between transects. *Scomber japonicus* also played a key role in distinguishing Durban and Thukela transects from Aliwal, iSimangaliso and Richards Bay transects, contributing 14–29% to dissimilarity.

## Discussion

This study presents a regional-scale synoptic account of ichthyoplankton along the east coast of South Africa with a particular focus on their distribution in relation to the contrasting environment of the Natal Ecoregion with that of the adjacent, northern Delagoa Ecoregion, which is more tropical. We used metabarcoding to identify ichthyoplankton to species level followed by a community analysis exploring distributional patterns. The main aims of this study were to generate presence-absence matrices of ichthyoplankton species followed by post hoc verification; test for the presence of species from different adult habitats, ocean zones and distribution ranges; and explore patterns in ichthyoplankton community composition associated with prominent environmental characteristics of the region.

Metabarcoding detected 67 ichthyoplankton species with a >97% similarity to barcode reference sequences on BOLD and GenBank. The species detected by metabarcoding are an underestimate of the number of species that occur in the region, with more than 3000 species known from the WIO [59], and at least 2200 off southern Africa with a large percentage occurring off the KZN coast [50]. Despite the use of taxon-specific mini-barcode primers for ichthyoplankton to mitigate the quality and quantity of DNA [47, 60], species detection could have been influenced by an array of technical, biological, and environmental factors. The number of species detected is likely to have been supressed by low sampling intensity and the spatio-temporal variation in field sampling. All sampling took place over the KZN shelf, at depth soundings of ≤200 m (i.e., only shelf waters sampled). Therefore, ichthyoplankton present in the samples were limited to occurrences in surface and sub-surface waters at these depth soundings. Sampling took place during late winter to spring (August to December) which could have further influenced the species of ichthyoplankton present due to seasonality.

Previous studies carried out over the KZN shelf have indicated distinct assemblages of larvae occurring at different times of the year [32]. Differences have been linked to seasonal oceanographic conditions, showing clear spatial and temporal variation in distribution and abundance of ichthyoplankton [33, 34, 36]. Notably, several small pelagic fish species undergo annual northward winter migrations into subtropical KZN waters to spawn, correlating seasonality with spawning events and their effects on detection rates of highly abundant larvae [13, 29, 36, 61]. This partly explains the influential role of small pelagic fish species in characterising the communities of all areas and depths we sampled. The relatively higher levels of diversity we detected in the lowest latitude transect to the north likely reflects contributions from a more diverse tropical ichthyofauna [62]. It is expected that there would be an increase in species detection rates if sampling took place throughout the water column, extending further offshore into oceanic waters and all year round.

Incomplete reference libraries (BOLD and GenBank) for fishes are expected to further reduce opportunities for species detections using ichthyoplankton. For example, many species present in South African waters and throughout the world do not have corresponding barcode records available in online reference libraries, thus limiting the number of ASVs that can be identified to species level in samples. Consequently, continued foundational biodiversity research to link DNA barcodes to taxonomically validated species is required to overcome this hurdle in future metabarcoding projects, by matching a greater proportion of ASVs with validated species descriptions [9, 63].

Larvae and/or eggs originating from different adult habitats (pelagic and benthic), ocean zones (coastal, neritic, and oceanic) and distribution ranges (SWIO [endemic], broader WIO, IWP and circumglobal) were detected in the ichthyoplankton. The metabarcoding results confirmed the findings of an earlier study from the same region (but extending further offshore to the 2000 m depth isobath) which relied on visual identifications of fish eggs and larvae [35]. The latter study reported 130 teleost families from diverse adult habitats, ocean zones and distribution ranges. The presence of tropical ichthyoplankton species, and latitudinal change in community composition found in our study, can be explained by southwards dispersal of tropical species into the subtropical study area by the Mozambique Channel eddies and upper Agulhas Current, similarly reported for decapod crustaceans [64].

*Engraulis capensis*, *E*. *nitidus* and *B*. *pterotum* demonstrated this latitudinal relationship as they increased in frequency in a northwards direction, whereas *E*. *whiteheadi* showed the opposite relationship and increased in frequency southwards. The significant latitudinal relationship indicated by the change in the ichthyoplankton community composition likely reflects the transition between northerly tropical waters and more southerly temperate waters that is mirrored in the biogeography of adult coastal fishes on the east coast of South Africa [62]. Specifically, our study shows a marked difference in the ichthyoplankton community of the iSimangaliso area (Delagoa Ecoregion) in the north from the communities of the other areas further south, which fall into the Natal Ecoregion. Somewhat anomalously, *E*. *capensis* ichthyoplankton was most markedly characteristic of the northern iSimangaliso area. Historically, *E*. *capensis* adults were concentrated in the Agulhas Ecoregion off the southern Cape, with a recent northeastward range expansion into the Natal and Delagoa Ecoregions [65]. Next-most characteristic taxa of the iSimangaliso ichthyoplankton were *E*. *nitidus* and *B*. *pterotum*, which are widely distributed in the WIO [59]. Notably, only larvae of the myctophid *B*. *pterotum* are known from the KZN coast [66], with the nearest adults reported from Mozambique [67].

Ichthyoplankton most characteristic of the sampled areas in the Natal Ecoregion were of *E*. *whiteheadi*, *E*. *wongratanai*, *S*. *japonicus* and *T*. *delagoa*, adults of which are very abundant off KZN at times [68]. Adult *Etrumeus* are sympatric off KZN, with *E*. *wongratanai* having a predominantly tropical distribution, and *E*. *whiteheadi* more temperate [69], possibly accounting for the higher prevalence of the latter’s ichthyoplankton in the southern sampled stations. Adults of *T*. *delagoa* and *S*. *japonicus* also co-occur off KZN, the former being sub-/ tropical and the latter anti-tropical [59]. *Scomber japonicus* appears to have also increased its prevalence off KZN relative to earlier years [70], supported by our finding of its ichthyoplankton being characteristic of the Durban and Thukela areas. Of the remaining ichthyoplankton most characteristic of the Natal Ecoregion, *Pomadasys olivaceus* has adults which are ubiquitous and very abundant off KZN [71], while *S*. *knysnaensis* is much less well known—its prevalence in the plankton is noteworthy as it is reef-associated and does not shoal, unlike the other species characterizing the ichthyoplankton of the sampled areas.

No direct observational studies of the reproductive biology, including spawning seasonality, of any of these species off KZN could be found, and only dated and very infrequent surveys of the distribution and abundance of their adults are available. This hampered contextual interpretation of observed patterns, but the presence in the plankton of these species indicates that spawning of these species does occur locally, or at least in close proximity, corroborating this finding by, amongst others [13, 29, 36].

Although bottom depth did not explain a significant amount of variation in ichthyoplankton community composition in our study, it is probable that distance from the coast and distance from the shelf edge covaried enough with depth to reduce the magnitude by which depth alone could account for community variation in the hierarchical analysis. Also, distances between sampling stations within transects, apart from Thukela, were relatively small, so mixing of water and ichthyoplankton from the stations at different depth soundings would have been facilitated, given the dynamic oceanography to the north of Richards Bay and south of Durban [14]. Notably, distance from the shelf edge accounted for a significant amount of the variation in ichthyoplankton composition (most species were neritic/coastal), largely driven by *T*. *delagoa*, which increased in frequency with distance away from the shelf edge; i.e., there was a greater incidence in shallow shelf waters. This species prefers nearshore habitats although it can also be found in deeper waters [59].

Beckley (1995) found that the concentration of larvae decreased further offshore (1000 m and 2000 m isobaths), and that the composition of larval assemblages changed in relation to water depth [35]. Larvae of oceanic mesopelagic fishes (Myctophidae) were highly abundant, while clupeid and scombrid larvae were abundant over the shelf / shelf-edge, and perciform larvae important to fisheries were associated with shelf waters and generally not found offshore in the Agulhas Current. Dispersal of ichthyoplankton is affected by intrusions of the Agulhas Current water onto the shelf, facilitating onshore transport of larvae of oceanic species [35]. Beckley and van Ballegooyen (1992) listed mechanisms likely to entrain and retain larvae over the shelf, with Agulhas Current intrusions, minor upwellings, cyclonic eddies, changes in wind direction and current reversals contributing to shoreward movement of larvae [14].

Similarly, distance from the coast was included in our study to assess whether there were gradients in ichthyoplankton community composition related to life history strategies such as inshore-association, or affinities with estuaries and river mouths which are prominent in the Natal Ecoregion, especially the KZN Bight. Distance from the coast was found to account for a significant amount of ichthyoplankton community variation. *Scomber japonicus* was a key species driving this variation, increasing in frequency with increasing distance from the coast. While Beckley and Leis (2000) [36] found high *S*. *japonicus* inshore densities off KZN, these larvae also occurred around the shelf edge, congruent with the shelf and upper slope habits of the pelagic adults [72]. It is a broadcast spawner that produces many small eggs and larvae that form dense patches in the zooplankton during winter and spring (August to November) [13, 36]. Tow-net samples for the present study were collected between August and November 2018, thus overlapping the known spawning season of *S*. *japonicus*.

Prior to our study, the most recent ichthyoplankton research off KZN was by Collocott (2016), who collected 107 samples in January and July 2010 on the shelf (<100 m water depths), mid-slope (100 m– 600 m water depths) and deep-slope (~1000 m water depths) [32]. Myctophidae, Bregmacerotidae and Engraulidae were the most widely occurring taxa of the 68 families found. There was a strong seasonal correlation, with highest larval density occurring during the dry season (winter, July sample) and lowest during the wet season (summer, January sample). Collocott (2016) could, however, not resolve larval samples to species level, and her study further excluded fish eggs, which could be identified to species during metabarcoding in the present study [32].

In conclusion, our study highlights advancements towards establishing species-level resolution of ichthyoplankton communities in the Western Indian Ocean, using DNA metabarcoding. We further confirm previous observations that the sheltered KZN Bight is an important area for spawning of several fish species and for retention of their ichthyoplankton. The distribution of ichthyoplankton species along a latitudinal gradient, and associations with coastal and shelf-edge processes in the WIO region were novel findings, and reflect key attributes likely influencing community composition of adult fishes in the region.

## Supporting information

S1 Table Samples collected (n = 88) from the different transects (iSimangaliso, Richards Bay, Thukela, Durban (1 and 2), and Aliwal) at different depth soundings (20, 50, 100 m) using different net types (R = ring, M = manta, W = WP2). The number next to each net type indicates the number of replicates per net taken at each depth sounding. 20/30*—20 m was sampled at all the transects except at Richards Bay where only 30 m was sampled.
(PDF)

S2 Table High-throughput sequencing outputs for each of the depth soundings per transect. (PDF)

S3 Table Fish species detected by metabarcoding of ichthyoplankton collected over the continental shelf of eastern South Africa, and verification of adult distribution ranges, habitats, and occurrence in different ocean zones. Barcode records on the BOLD database were used for species identification. The % similarity to barcode sequences is shown for each species, as well as the accession numbers, geographical origin and number of sequences available on BOLD. The WoRMS, OBIS and GBIF online databases were consulted to confirm the distribution range (Endemic = Endemic to the southwest Indian Ocean; WIO = Western Indian Ocean, including north of the equator; IWP = Indo-West Pacific), habitat and ocean zone frequented by individual species.
(PDF)

S4 Table Summary statistics of the various environmental variables associated with each plankton sample. SD = standard deviation. * = days. The average ± standard deviation latitude of samples was -29.6329 ± 0.6902°, with the southern- and northern-most samples extending from -30.2886° to -27.9202°, respectively. Longitude averaged 31.3772 ± 0.5568° and ranged by 1.8°. The average isobath sampled was 87.5 ± 66.8 m. Average distance to coast and distance to shelf edge of samples was 10.2 ± 12.0 km and 7.3 ± 10.1 km, respectively. The average date of sampling was in the austral spring of 2018, and samples were collected within a range of 369 days.
(PDF)
