# Supplementary material for: Metabarcoding of ichthyoplankton communities associated with a highly dynamic shelf region of the southwest Indian Ocean
Source: PLoS One. 2023 Apr 27;18(4):e0284961. doi: 10.1371/journal.pone.0284961 (PMC10138858; doi:10.1371/journal.pone.0284961)
Supplement: S1 Table — The number next to each net type indicates the number of replicates per net taken at each depth sounding. 20/30*—20 m was sampled at all the transects except at Richards Bay where only 30 m was sampled. (PDF) [file pone.0284961.s001.pdf]

**S1 Table. Samples collected (n = 88) from the different transects (iSimangaliso, Richards Bay, Thukela, Durban (1 and 2), and Aliwal) at different depth soundings (20, 50, 100 m) using different net types (R = ring, M = manta, W= WP2).** The number next to each net type indicates the number of replicates per net taken at each depth sounding. 20/30* - 20 m was sampled at all the transects except at Richards Bay where only 30 m was sampled.

| **Depth** | **iSimangaliso** | **Richards Bay** | **Thukela** | **Durban 1** | **Durban 2** | **Aliwal** |
|---|---|---|---|---|---|---|
| **20/30*** | 1R, 1M, 1W | 2R | 3R, 1M, 1W | 3R, 1W | 3R, 1M, 1W | 3R, 1M, 1W |
| **50** | 1R, 1M, 1W | - | 3R, 1M, 1W | 2R, 1M, 1W | 3R, 1M, 1W | 3R, 1M, 1W |
| **100** | 1R, 1M, 1W | - | 3R, 1M, 1W | 3R, 1M, 1W | 3R, 1M, 1W | 3R, 1M, 1W |
| **200** | - | - | 3R, 1M, 1W | 2R, 1M, 1W | 3R, 1M, 1W | 3R, 1M, 1W |
