# Supplementary material for: Metabarcoding of ichthyoplankton communities associated with a highly dynamic shelf region of the southwest Indian Ocean
Source: PLoS One. 2023 Apr 27;18(4):e0284961. doi: 10.1371/journal.pone.0284961 (PMC10138858; doi:10.1371/journal.pone.0284961)
Supplement: S2 Table — (PDF) [file pone.0284961.s002.pdf]

S2 Table. High-throughput sequencing outputs for each of the depth soundings per transect.

Library	Depth	Read count	Merged reads	Total amplicon sequence variants	Total amplicon sequence variants of ichthyoplankton	Total merged amplicon sequence variants	Merged amplicon sequence variants assigned to species level
**iSimangaliso**	20	797208	19745	250	23	16	13
	50	745330	25241	206	22	13	11
	100	601588	16896	191	32	15	11
**Richards bay**	30	1391991	45588	149	7	6	6
**Thukela**	20	226444	17173	76	24	7	7
	50	250990	9817	73	11	3	3
	100	330306	22734	93	41	10	6
	200	238170	12742	105	26	11	10
**Durban 2018**	20	658916	8539	88	14	6	6
	50	347234	9710	95	11	4	2
	100	600746	21750	133	15	6	6
	200	345572	15390	113	29	14	14
**Durban 2019**	20	869278	5304	74	10	7	5
	50	633810	9463	50	7	4	4
	100	619826	32631	292	23	8	4
	200	644274	48199	311	25	15	10
**Aliwal**	20	60446	6370	152	9	8	8
	50	50792	5854	187	13	10	7
	100	31910	5225	144	13	9	9
	200	44358	8377	192	27	15	14
**Total across all sites**	-	9 489 189	346 748	1726	219	90	67
