# Supplementary material for: Metabarcoding of ichthyoplankton communities associated with a highly dynamic shelf region of the southwest Indian Ocean
Source: PLoS One. 2023 Apr 27;18(4):e0284961. doi: 10.1371/journal.pone.0284961 (PMC10138858; doi:10.1371/journal.pone.0284961)
Supplement: S3 Table — Barcode records on the BOLD database were used for species identification. The % similarity to barcode sequences is shown for each species, as well as the accession numbers, geographical origin and number of sequences available on BOLD. The WoRMS, OBIS and GBIF online databases were consulted to confirm the distribution range (Endemic = Endemic to the southwest Indian Ocean; WIO = Western Indian Ocean, including north of the equator; IWP = Indo-West Pacific), habitat and ocean zone frequented by individual species. (PDF) [file pone.0284961.s003.pdf]

S3 Table. Fish species detected by metabarcoding of ichthyoplankton collected over the continental shelf of eastern South Africa, and verification of adult distribution ranges, habitats, and occurrence in different ocean zones. Barcode records on the BOLD database were used for species identification. The % similarity to barcode sequences is shown for each species, as well as the accession numbers, geographical origin and number of sequences available on BOLD. The WoRMS, OBIS and GBIF online databases were consulted to confirm the distribution range (Endemic = Endemic to the southwest Indian Ocean; WIO = Western Indian Ocean, including north of the equator; IWP = Indo-West Pacific), habitat and ocean zone frequented by individual species.

No.	Order	Family	Species	% similarity	BIN (BOLD)	Records (n), location collected	Known distribution range	Datasets consulted (WoRMS, GBIF, OBIS, etc)	Adult habitats	Ocean zone	Distribution range
**1**	Labriformes	Labridae	*Anampses caeruleopunctatus*	100	BOLD:AAB9969	(55) South Africa, Indonesia	WIO, South Africa	OBIS; GBIF	Benthic-reef	Neritic_inshore	IWP
**2**	Kurtiformes	Apogonidae	*Apogon semiornatus*	100	BOLD:AAD2206	(25) South Africa, Madagascar	WIO, South Africa	OBIS; GBIF	Benthic-reef	Neritic_inshore	IWP
**3**	Scombriformes	Ariommatidae	*Ariomma indicum (indica)*	100	BOLD:ACF5823; BOLD:AAB5941	(86) South Africa, India	WIO, South Africa	OBIS; GBIF	Benthopelagic	Neritic	IWP
**4**	Myctophiformes	Myctophidae	*Benthosema pterotum*	99.02	BOLD:AAM9344	(25) Indonesia	WIO, South Africa	OBIS; GBIF	Mesopelagic	Oceanic	IWP
**5**	Myctophiformes	Myctophidae	*Benthosema suborbitale*	100	BOLD:AAI8542	(31) Cayman Island	WIO, South Africa	OBIS; GBIF	Mesopelagic	Oceanic	Circumglobal
**6**	Perciformes	Caesionidae	*Caesio caerulaurea*	100	BOLD:ABZ2197	(87) WIO, Indonesia	WIO, South Africa	OBIS; GBIF	Epipelagic	Neritic_inshore	IWP
**7**	Callionymiformes	Callionymidae	*Callionymus marleyi*	99,67	BOLD:AAW8707	(7) South Africa	WIO, South Africa	OBIS; GBIF	Benthic-soft	Neritic_inshore	WIO
**8**	Beryciformes	Berycidae	*Centroberyx spinosus*	100	BOLD:AAF8778	(10) South Africa	South Africa	OBIS; GBIF	Benthopelagic	Neritic	Endemic
**9**	Scombriformes	Centrolophidae	*Centrolophus niger*	100	BOLD:AAC3522	(11) South Africa	WIO, South Africa	OBIS; GBIF	Mesopelagic	Oceanic	Circumglobal
**10**	Ovalentaria	Pomacentridae	*Chromis dasygenys*	100	BOLD:AAJ5401	(3) South Africa	WIO, South Africa	OBIS; GBIF	Benthic-reef	Neritic_inshore	Endemic
**11**	Ovalentaria	Pomacentridae	*Chromis opercularis / Chromis weberi*	100	BOLD:ACF0042	(13) South Africa, Mozambique	WIO, South Africa	OBIS; GBIF	Benthic-reef	Neritic_inshore	WIO
**12**	Scorpaeniformes	Platycephalidae	*Cociella heemstrai*	97,52	BOLD:AAD7871	(24) South Africa	WIO, South Africa	OBIS; GBIF	Benthic-soft	Neritic	WIO

**13**	Scombriformes	Nomeidae	*Cubiceps whiteleggii*	99,51	BOLD:AAB5183	(63) South Africa, Australia	WIO, South Africa	OBIS; GBIF	Mesopelagic	Oceanic	IWP
**14**	Stomiiformes	Gonostomatidae	*Cyclothone acclinidens*	100	BOLD:AAU0299	(29) Atlantic Ocean	WIO, South Africa	OBIS; GBIF	Mesopelagic	Oceanic	Circumglobal
**15**	Stomiiformes	Gonostomatidae	*Cyclothone microdon*	100	BOLD:AAB4944	(50) Canada	WIO, South Africa	OBIS; GBIF	Mesopelagic	Oceanic	Circumglobal
**16**	Pleuronectiformes	Cynoglossidae	*Cynoglossus lida*	100	BOLD:AAC0744; BOLD:AAB8676	(49) South Africa	WIO, South Africa	OBIS; GBIF	Benthic-soft	Neritic	IWP
**17**	Carangiformes	Carangidae	*Decapterus macarellus*	100	BOLD:AAC4792	(245) South Africa, Indonesia, Philippines	WIO, South Africa	OBIS; GBIF	Epipelagic	Neritic	Circumglobal
**18**	Carangiformes	Carangidae	*Decapterus macrosoma*	100	BOLD:ADI4344	(334) South Africa, Malaysia, Philippines	WIO, South Africa	WoRMS; OBIS; GBIF	Epipelagic	Neritic	IWP
**19**	Carangiformes	Carangidae	*Decapterus russelli*	100	BOLD:AAB6796	(154) South Africa	WIO, South Africa	WoRMS; OBIS; GBIF	Epipelagic	Neritic	IWP
**20**	Myctophiformes	Myctophidae	*Diaphus richardsoni*	100	BOLD:AAE0839	(31) Australia, Indian Ocean	WIO, South Africa	OBIS; GBIF	Mesopelagic	Oceanic	Circumglobal
**21**	Perciformes	Caesionidae	*Dipterygonotus balteatus*	100	BOLD:AAD3666	(36) South Africa, Indonesia	WIO, South Africa	OBIS; GBIF	Epipelagic	Neritic	IWP
**22**	Acanthuriformes	Emmelichthyidae	*Emmelichthys nitidus*	100	BOLD:AAE9975; BOLD:AAE9976	(63) South Africa, Australia	South Africa, Pacific Ocean	OBIS; GBIF	Benthopelagic	Neritic	Circumglobal (S)
**23**	Clupeiformes	Engraulidae	*Encrasicholina punctifer*	100	BOLD:AAF8837	(124) South Africa, China	WIO, South Africa	OBIS; GBIF	Epipelagic	Neritic	IWP
**24**	Clupeiformes	Engraulidae	*Engraulis capensis*	100	BOLD:AAB2317	(162) South Africa, China	WIO, South Africa	OBIS; GBIF	Epipelagic	Neritic	WIO
**25**	Clupeiformes	Clupeidae	*Etrumeus whiteheadi*	100	BOLD:AAC3892	(21) South Africa	WIO, South Africa	OBIS; GBIF	Epipelagic	Neritic	WIO
**26**	Clupeiformes	Clupeidae	*Etrumeus wongratanai*	100	BOLD:AAC2444	(28) South Africa	WIO, South Africa	GBIF	Epipelagic	Neritic	WIO
**27**	Labriformes	Labridae	*Halichoeres cosmetus*	100	BOLD:AAC1194	(16) South Africa	WIO, South Africa	OBIS; GBIF	Benthic-reef	Neritic_inshore	IWP
**28**	Myctophiformes	Myctophidae	*Hygophum hygomii*	100	BOLD:AAC6495	(63) Atlantic Ocean, Indian Ocean	WIO, South Africa	OBIS; GBIF	Mesopelagic	Oceanic	Circumglobal
**29**	Myctophiformes	Myctophidae	*Lampanyctus australis*	100	BOLD:AAC0460	(49) Atlantic Ocean, Tasman Sea	WIO, South Africa	OBIS; GBIF	Mesopelagic	Oceanic	Circumglobal (S)

**30**	Myctophiformes	Myctophidae	*Lampanyctus lepidolychnus*	99,67	BOLD:AAB3777	(42) South Africa, Canada	South Africa	OBIS; GBIF	Mesopelagic	Oceanic	Circumglobal (S)
**31**	Myctophiformes	Myctophidae	*Lobianchia gemellarii*	99,67	BOLD:AAC7797	(36) Atlantic Ocean, Indian Ocean	WIO, South Africa	OBIS; GBIF	Mesopelagic	Oceanic	Circumglobal
**32**	Aulopiformes	Notosudidae	*Luciosudis normani*	100	BOLD:AAK6733	(6) New Zealand	WIO, South Africa	OBIS; GBIF	Mesopelagic	Oceanic	Circumglobal (S)
**33**	Stomiiformes	Sternoptychidae	*Maurolicus walvisensis*	100	BOLD:AEH5284	(40) Atlantic Ocean, Indian Ocean	South Africa	OBIS; GBIF	Mesopelagic	Oceanic	WIO
**34**	Perciformes	Monodactylidae	*Monodactylus argenteus*	100	BOLD: AAA9698; BOLD:AAA9699	(81) SouthAfrica, Madagascar	WIO, South Africa	OBIS; GBIF	Epipelagic	Neritic_inshore	IWP
**35**	Perciformes	Monodactylidae	*Monodactylus falciformis*	100	BOLD:AAE4819	(16) South Africa	WIO, South Africa	OBIS; GBIF	Epipelagic	Neritic_inshore	IWP
**36**	Myctophiformes	Myctophidae	*Myctophum selenops*	99,51	BOLD:AAU3494	(27) Indian Ocean	WIO, South Africa	OBIS; GBIF	Mesopelagic	Oceanic	Circumglobal
**37**	Scombriformes	Gempylidae	*Nealotus tripes*	96,33	BOLD:AAC6290	(40) Canada	WIO, South Africa	OBIS; GBIF	Mesopelagic	Oceanic	Circumglobal
**38**	Myctophiformes	Myctophidae	*Notolychnus valdiviae*	100	BOLD:AAF2698	(40) Atlantic Ocean, Pacific Ocean	WIO, South Africa	OBIS; GBIF	Mesopelagic	Oceanic	Circumglobal
**39**	Anguilliformes	Ophichthidae	*Ophisurus serpens*	99,51	BOLD:AAE1881; BOLD:ADO0383	(27) South Africa, Indonesia	WIO, South Africa	OBIS; GBIF	Benthic-soft	Neritic	IWP
**40**	Blenniiformes	Blenniidae	*Parablennius pilicornis*	100	BOLD:AAF7833	(16) South Africa	South Africa	OBIS; GBIF	Benthic-reef	Neritic_inshore	WIO
**41**	Labriformes	Labridae	*Paracheilinus mccoskeri*	100	BOLD:AAE2895	(12) Sri Lanka	WIO	OBIS	Benthic-reef	Neritic_inshore	IWP
**42**	Trachiniformes	Pinguipedidae	*Parapercis somaliensis*	98,99	BOLD:AAI5793	(1) South Africa	WIO	OBIS; GBIF	Benthic-soft	Neritic	WIO
**43**	Perciformes	Mullidae	*Parupeneus fraserorum*	99,51	BOLD:AAF8776	(31) South Africa	WIO, South Africa	OBIS; GBIF	Benthic-soft	Neritic	Endemic
**44**	Perciformes	Haemulidae	*Plectorhinchus flavomaculatus*	100	BOLD:AAC4020	(27) SouthAfrica, Madagascar	WIO, South Africa	WoRMS; OBIS; GBIF	Benthic-reef	Neritic_inshore	IWP
**45**	Perciformes	Haemulidae	*Pomadasys olivaceus*	100	BOLD:AAD1379	(42) South Africa	WIO, South Africa	OBIS; GBIF	Benthic-soft	Neritic_inshore	WIO
**46**	Perciformes	Haemulidae	*Pomadasys striatus*	100	BOLD:AAD1385	(19) South Africa	WIO, South Africa	OBIS; GBIF	Benthic-reef	Neritic_inshore	Endemic
**47**	Mugiliformes	Mugilidae	*Pseudomyxus capensis*	100	BOLD:AAC5938	(21) South Africa	South Africa	OBIS; GBIF	Benthic-soft	Neritic_inshore	Endemic
**48**	Pleuronectiformes	Paralichthyidae	*Pseudorhombus elevatus*	100	BOLD:AAB6447; BOLD:AAB6448	(36) South Africa	WIO, South Africa	OBIS; GBIF	Benthic-soft	Neritic	IWP

**49**	Kurtiformes	Apogonidae	*Rhabdamia gracilis*	100	BOLD:AAC9583	(33) China	WIO, South Africa	OBIS; GBIF	Benthic-reef	Neritic_inshore	IWP
**50**	Clupeiformes	Clupeidae	*Sardinella gibbosa*	100	BOLD:AAB7263; BOLD:AAB7262	(201) Israel, Thailand, Mozambique	WIO, South Africa	WORMS; OBIS; GBIF	Epipelagic	Neritic	IWP
**51**	Clupeiformes	Clupeidae	*Sardinops sagax*	99,67	BOLD:AAB6180	(141) South Africa, Australia	WIO, South Africa	WoRMS; OBIS; GBIF	Epipelagic	Neritic	Circumglobal
**52**	Aulopiformes	Synodontidae	*Saurida cf undosquamis*	100	BOLD:AAB1853; BOLD:ACG7154; BOLD:AAD1070; BOLD:AAB1852; BOLD:AAB1856	(170) South Africa, Australia, India	WIO, South Africa	OBIS; GBIF	Benthic-soft	Neritic	WIO
**53**	Blenniiformes	Blenniidae	*Scartella emarginata*	100	BOLD:AAE4361	(9) South Africa	WIO, South Africa	OBIS; GBIF	Benthic-reef	Neritic_inshore	IWP
**54**	Scombriformes	Scombridae	*Scomber japonicus*	100	BOLD:AAA5957	(227) South Africa, Turkey	WIO, South Africa	OBIS; GBIF	Epipelagic	Neritic	IWP
**55**	Myctophiformes	Myctophidae	*Scopelopsis multipunctatus*	100	BOLD:AAD1092	(26) South Africa	WIO, South Africa	OBIS; GBIF	Mesopelagic	Oceanic	Circumglobal (S)
**56**	Aulopiformes	Notosudidae	*Scopelosaurus hamiltoni*	100	BOLD:AAI0783; BOLD:AAI0784	(6) Australia	South Africa	OBIS; GBIF	Mesopelagic	Oceanic	Circumglobal (S)
**57**	Perciformes	Serranidae	*Serranus cabrilla*	100	BOLD:AAD1027	(128) Turkey	WIO, South Africa	OBIS; GBIF	Benthic-reef	Neritic	Endemic
**58**	Spariformes	Sillaginidae	*Sillago sihama*	100	BOLD:AAA7603	(5) South Africa	WIO	GBIF	Benthic-soft	Neritic_inshore	IWP
**59**	Scorpaeniformes	Platycephalidae	*Sorsogona portuguesa*	100	BOLD:AAF8784	(21) South Africa	WIO, South Africa	OBIS; GBIF	Benthic-soft	Neritic	Endemic
**60**	Istiophoriformes	Sphyraenidae	*Sphyraena chrysotaenia*	100	BOLD:AAD0400	(61) South Africa, Mozambique	WIO, South Africa	OBIS; GBIF	Epipelagic	Neritic	IWP
**61**	Spariformes	Sparidae	*Spicara australis*	100	BOLD:AAO4385	(4) South Africa	WIO, South Africa	OBIS; GBIF	Mesopelagic	Oceanic	Endemic
**62**	Spariformes	Sparidae	*Spondyliosoma emarginatum*	100	BOLD:ABZ6175	(13) South Africa	WIO, South Africa	OBIS; GBIF	Benthic-reef	Neritic_inshore	Endemic
**63**	Labriformes	Labridae	*Stethojulis albovittata*	100	BOLD:AAD4824	(31) Mozambique, Reunion	WIO, South Africa	OBIS; GBIF	Benthic-reef	Neritic_inshore	WIO
**64**	Stomiiformes	Stomiidae	*Stomias boa*	99,67	BOLD:AAB1180	(86) South Africa, Canada	South Africa	OBIS; GBIF	Mesopelagic	Oceanic	Circumglobal

**65**	Carangiformes	Carangidae	*Trachurus delagoa*	100	BOLD:AAA8 614	(55) South Africa	WIO, South Africa	OBIS; GBIF	Epipelagic	Neritic	Endemic
**66**	Acanthuriformes	Sciaenidae	*Umbrina robinsoni /Umbrina canariensis*	100	BOLD:AAE0 529	(12) South Africa	South Africa	OBIS; GBIF	Benthic-reef	Neritic	WIO
**67**	Perciformes	Mullidae	*Upeneus guttatus*	100	BOLD:AAB9 327	(113) Indonesia, Vietnam	WIO, South Africa	OBIS; GBIF	Benthic-soft	Neritic	IWP
