# Supplementary material for: Metabarcoding of ichthyoplankton communities associated with a highly dynamic shelf region of the southwest Indian Ocean
Source: PLoS One. 2023 Apr 27;18(4):e0284961. doi: 10.1371/journal.pone.0284961 (PMC10138858; doi:10.1371/journal.pone.0284961)
Supplement: S4 Table — SD = standard deviation. * = days. The average ± standard deviation latitude of samples was -29.6329 ± 0.6902°, with the southern- and northern-most samples extending from -30.2886° to -27.9202°, respectively. Longitude averaged 31.3772 ± 0.5568° and ranged by 1.8°. The average isobath sampled was 87.5 ± 66.8 m. Average distance to coast and distance to shelf edge of samples was 10.2 ± 12.0 km and 7.3 ± 10.1 km, respectively. The average date of sampling was in the austral spring of 2018, and samples were collected within a range of 369 days. (PDF) [file pone.0284961.s004.pdf]

S4 Table. Summary statistics of the various environmental variables associated with each plankton sample. SD = standard deviation. * = days. The average ± standard deviation latitude of samples was -29.6329 ± 0.6902°, with the southern- and northern-most samples extending from -30.2886° to -27.9202°, respectively. Longitude averaged 31.3772 ± 0.5568° and ranged by 1.8°. The average isobath sampled was 87.5 ± 66.8 m. Average distance to coast and distance to shelf edge of samples was 10.2 ± 12.0 km and 7.3 ± 10.1 km, respectively. The average date of sampling was in the austral spring of 2018, and samples were collected within a range of 369 days.

Statistic	Latitude (°)	Longitude (°)	Isobath (m)	Dist_coast (km)	Dist_shelf (km)	Date
Average	-29.6329	31.3772	87.5	10.2	7.3	2018/11/23
SD	0.6902	0.5568	66.8	12.0	10.1	132*
Min	-30.2886	30.8033	20.0	1.1	0.0	2018/08/02
Max	-27.9202	32.6132	200.0	42.7	39.5	2019/08/06
